# Cell- and region-specific miR30-based gene knock-down with temporal control in the rat brain

**DOI:** 10.1186/1471-2199-11-93

**Published:** 2010-12-06

**Authors:** Beihui Liu, Haibo Xu, Julian FR Paton, Sergey Kasparov

**Affiliations:** 1 Department of Physiology and Pharmacology, Bristol Heart Institute, School of Medical Sciences, University of Bristol, UK; 2 Department of Pharmacology, State Key Laboratory for Research and Development of Chinese Materia Medica, Chengdu University of Traditional Chinese Medicine, Chengdu 611137, P.R. China

## Abstract

**Background:**

RNA interference (RNAi) emerges as a powerful tool to induce loss-of-function phenotypes. In the context of the brain, gene manipulation is best targeted to specific subsets of cells in order to achieve a physiologically relevant outcome. Polymerase II-based viral expression systems can be used to cell-specifically express constructs incorporating flanking and loop sequences from endogenous microRNA (miRNA), which directs the designed hairpins into the endogenous gene silencing machinery. While many studies have documented non-cell-selective gene knock-down in the brain, it has not been tested whether different cell types or different areas of the central nervous system (CNS) are equally amenable to this approach. We have evaluated this issue using a tetracycline (Tet)-controllable and cell-specific miRNA 30 (miR30)-based short hairpin (shRNA) interference system.

**Results:**

To achieve targeted expression two cell type-specific promoters were used; the enhanced compact glial fibrillary acidic protein (GfaABC_1_D) promoter and the enhanced human synapsin-1 (SYN) promoter. Powerful luciferase (Luc) and the neuronal isoform of nitric oxide synthase (nNOS) gene knock-down were achieved both *in vitro *and *in vivo*. Administration of doxycycline (Dox) abrogated gene silencing. However, the efficacy of gene knock-down in both neurones and astrocytes in the hippocampus (HIP) was lower than that in the dorsal vagal complex of the brainstem (DVC). This was not due to regional differences in the expression of the the key enzymes involved in miRNA processing.

**Conclusions:**

The results from the presented experiments demonstrated that selective gene knock-down in subsets of brain cells is achievable. However, there are some presently unknown regional factors which affect either the processing of miRNA-based cassettes or their potency for gene silencing.

## Background

RNAi is a conserved regulatory process that mediates sequence-specific post-transcriptional gene silencing in a variety of species [1,2]. The RNAi technique has become a potent experimental tool for functional analysis of genes and is believed to have significant therapeutic potential [3-6]. In the context of the brain, where numerous cell types with different functions are intermixed, gene knock-down targeted to a cellular specified phenotype in most cases would be preferable, in order to achieve a physiologically or medically relevant outcome [7,8]. Within vector-based RNAi systems, miRNA-based cassettes, in which shRNA is embedded in a precursor miRNA context, are particularly attractive [9-12]. Endogenous miRNAs are transcribed predominantly by polymerase II (Pol II) promoters as long primary miRNAs transcripts (pri-miRNAs) that are subsequently processed into precursor miRNAs (pre-miRNAs) and eventually incorporated into the RNA-induced silencing complex (RISC) to mediate silencing of the target gene ([9]). Vectors can be made to mimic naturally occurring miRNAs whereby a designed pre-miRNA is embedded in an authentic miRNA context enabling its entry into the endogenous miRNA/RNAi pathway [9,11,13,14]. The value of this approach was demonstrated by Stegmeier *et al *who achieved an effective knock-down with only a single viral genome per cell [15]. This is an important feature given that it is hard to achieve high copy numbers in a single cell in the brain. Moreover, large libraries of pre-designed constructs of this type are commercially available from OpenBiosystems and Invitrogen. Typically, in these constructs the miRNA-like motifs are fused to the end of a sequence encoding a fluorescent protein, giving rise to a transcript which encodes both the pre-miRNA and the reporter gene. As this is a Pol II- driven system it can be controlled by a tetracycline (or its analogue Dox)-responsive promoter element (Tre) as used in many viral systems [16-18]. If expression of the transactivator such as the Tet-off transactivator (tTA) is cell type specific, this would allow targeting gene knock-down to a specific cell type. As the same gene frequently co-exists in both neurones and astrocytes in the brain and may have different or even opposite functions, cell-selective gene manipulation has obvious advantages. In addition, for most experimental and potential clinical applications, viral gene knock-down might be most advantageous if targeted to a selected brain region(s). It is usually assumed that the mechanisms of miRNA-mediated gene knock-down are ubiquitous and operate constitutively in various tissues [3,19-21]. However, concerning the brain, it has not yet been tested whether the same gene knock-down cassette is equally effective in different areas of the central nervous system (CNS).

There are two main objectives for this study: Firstly, we asked whether cell-specific and Dox-controllable gene knock-down can be achieved in both astrocytes and neurones. Secondly, we compared the efficacy of gene knock-down in two very different areas of the CNS, an evolutionary "old" part of the brain within the medulla oblongata involved with the control of various autonomic functions and a much "younger" part of the brain, the hippocampus (HIP). To this end, we constructed a binary Dox-controllable and cell-specific miR30-based RNAi system to express shRNAs targeting a reporter gene for Luc and an endogenous gene for nNOS. Luc was chosen as it allowed a quantifiable assessment of the efficacy of gene knock-down at the protein level. Furthermore, Luc quantification is reliable and technically straightforward. nNOS was chosen as a typical neuronal protein which is expressed in both areas of the brain we chose to study at moderate levels [22-25]. In these constructs gene targeting sequences were embedded in the precursor miRNA context derived from miR30, one of the most well-studied miRNA in mammals. This construct was fused to the 3' end of the GFP sequence and placed under control of the Tre promoter within a lentiviral vector (LVV) backbone. To drive its expression in a cell-specific manner it was co-applied with the other LVV expressing Tet-off transactivator tTA using either GfaABC_1_D promoter for astrocytes or SYN promoter for neurones, both promoters were transcriptionally enhanced using previously published approaches [26]. This yielded in a binary system which was both, cell-specific and Dox-controllable.

We demonstrate that significant knock-down can be achieved in a cell-selective and Dox-controllable manner for both an exogenous and endogenous targeted gene. However, there are major regional differences in the efficacy of the same gene knock-down cassette, which cannot be explained by the differential expression of the main known proteins involved in miRNA processing.

## Methods

### LVVs used in this study

**Figure 1:**
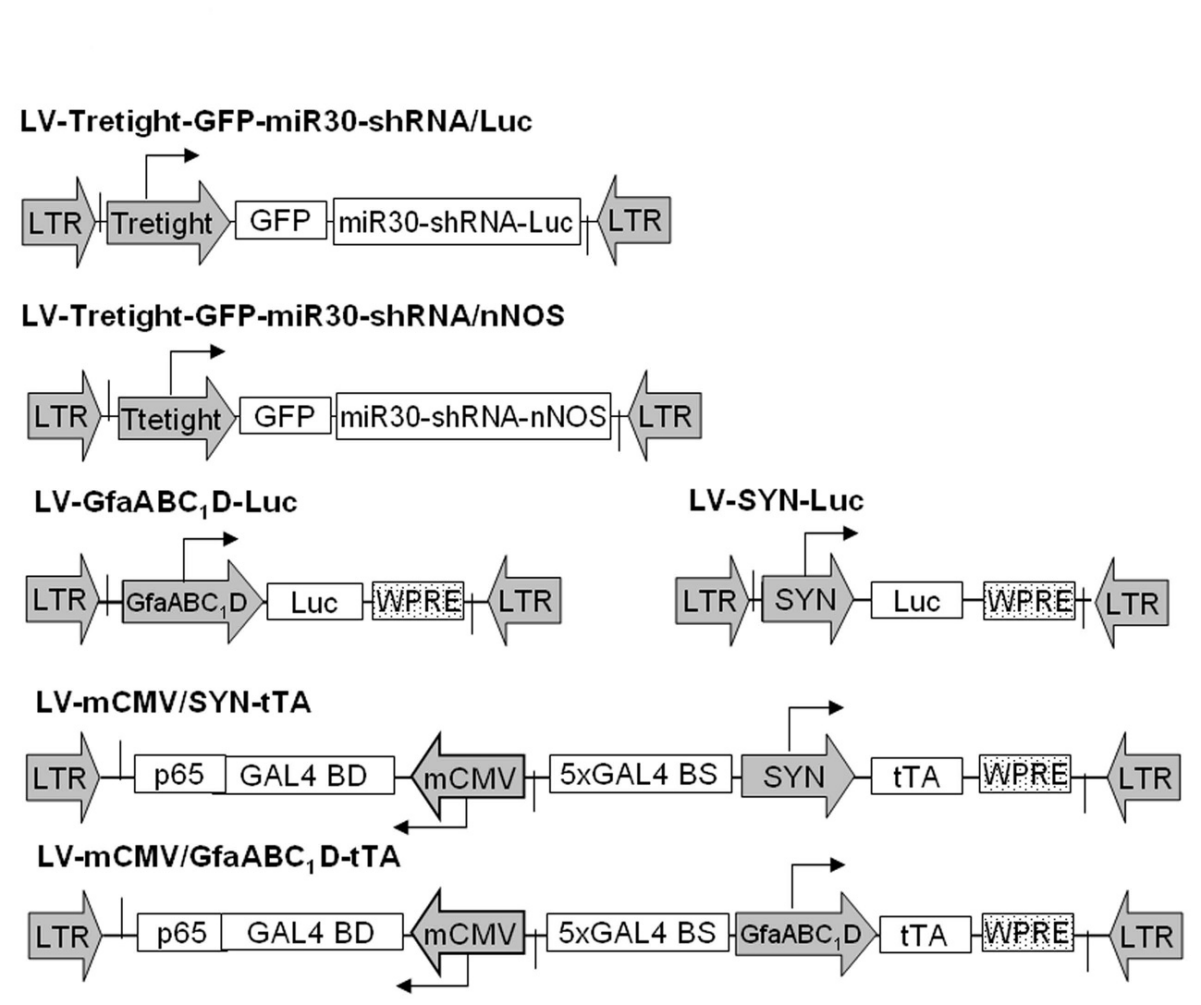
**Schematic representation of lentiviral vectors used in the current study**. LTR, lentiviral long terminal repeats; Tretight, a modified tetracycline and Dox-responsive promoter derived from pTRE-tight (Clontech); GFP, green fluorescence protein; miR30-shRNA/Luc, miR30-based shRNA targeting firefly Luc gene; miR30-shRNA/nNOS, miR30-based shRNA targeting rat neuronal nitric oxide synthase gene; Luc, firefly Luc gene; GfaABC_1_D, a compact glial fibrillary acidic protein promoter (690 bp); SYN, human synapsin 1 promoter (470 bp); mCMV, minimal CMV core promoter (65 bp); GAL4BDp65, a chimeric transactivator consisting of a part of the transactivation domain of the murine NF-κBp65 protein fused to the DNA binding domain of GAL4 protein from yeast; WPRE, woodchuck hepatitis post-transcriptional regulatory element.

Six LVVs were constructed for this study (Figure 1). (1) LV-Tretight-GFP-miR30-shRNA/Luc where the GFP sequence is followed by a miR30-embedded anti-Luc hairpin under the control of the Tretight promoter; (2) LV-Tretight-GFP-miR30-shRNA/nNOS, in which the GFP sequence is followed by a miR30-embedded anti-nNOS hairpin under the control of the Tretight promoter; (3) LV-GfaABC_1_D-Luc where the Luc gene is expressed by the astrocytic GfaABC_1_D promoter; (4) LV-SYN-Luc, in which the Luc gene is expressed by the neuronal SYN promoter; (5) LV-mCMV/SYN-tTA where the tTA gene expression is under the control of the enhanced SYN promoter by the bidirectional transcriptional amplification strategy [26]; and (6) LV-mCMV/GfaABC_1_D-tTA in which the tTA gene expression is under the control of the enhanced GfaABC_1_D promoter by the bidirectional transcriptionally amplification strategy. The Luc targeting shRNA sequence was provided by Dr. M.Z. Li (Howard Hughes Medical Institute, Boston) and the nNOS targeting sequence was designed by using the RNAi design algorithm at http://katahdin.cshl.org/siRNA/RNAi.cgi?type=shRNA and kindly provided by Dr. Luna Benvenisti (Zarom Teva Pharmaceutical Industries, USA). LV-GfaABC_1_D-Luc and LV-SYN-Luc served as sources of Luc in either an astrocyte- or neurone-specific manner. Additionally LV-SYN-WPRE and LV-GfaABC1D-WPRE (unpublished constructs in our lab, not illustrated) worked as control vectors to balance the total load of viral particles into cells or animal tissues.

### Plasmid construction

All the LVV plasmids were based on the improved lentiviral shuttle vector pTYF-SW-Linker backbone using standard cloning procedures [27]. To generate the LV-Tretight-GFP-miR30-shRNA/Luc shuttle vector, we excised the Tretight fragment containing the modified Tet-responsive promoter from pTRE-Tight-DsRed2 (Clontech) and inserted it into the pTYF-SW Linker and cloned, into the obtained vector, PCR product of GFP-miR30-shRNA/Luc cassette from pPRIME-CMV-GFP-FF3 (kindly provided by F. Stegmeier, Harvard Medical School) downstream of Tretight promoter. To construct the LV-Tretight-GFP-miR30-shRNA/nNOS shuttle vector, we replaced the Luc shRNA sequence in the LV-Tretight-GFP-miR30-shRNA/Luc shuttle vector with the nNOS shRNA. We obtained LV-Gfa ABC_1_D-Luc and LV-SYN-Luc shuttle vectors by replacing EGFP in pTYF-1×GfaABC_1_D-EGFP and pTYF-1×SYN-EGFP [26] with the Luc cDNA from the pGL3-Basic vector (Promega) respectively. LV-mCMV/GfaABC_1_D-tTA and LV-mCMV/SYN-tTA shuttle vectors were both produced by replacing EGFP in pTYF-mCMV/SYN-EGFP and pTYF-mCMV/GfaABC_1_D-EGFP [26] with the tTA cDNA from the LV-1×SYN-tTA shuttle vector [7].

### LVV production

The LVV system used in this study is derived from HIV-1 and pseudotyped with the vesicular stomatitis virus coat [28]. LVV stocks were produced by transient co-transfection of the shuttle plasmids, the packaging vector pNHP, and the envelope plasmid pHEF-VSVG in HEK293FT cells. Viral concentration and titration were carried out as described earlier [28].

### Cell culture and in vitro LVV vector transduction

The *in vitro *transduction experiments were carried out in a neurone-derived rat pheochromocytoma PC12 cell line and a 1321N1 glial cell line from human brain astrocytoma. PC12 cells were grown in Dulbecco's modified Eagle's medium (DMEM) supplemented with 10% heat-inactivated fetal bovine serum (FBS) and 5% horse serum. 1321N1 cells were cultured in DMEM supplemented with 10% heat-inactivated FBS. The cells were split and plated in 24-well plates at a cell density of 5 × 10^4 ^per well with 0.5 ml culture medium. After 24 h, cells were transduced overnight with appropriate LVVs in the presence of polybrene (8 μg/ml). The ratio for three vectors in all cotransfections was fixed to 1:1:1 and the total viral MOI per well was fixed to 5. Cells were then washed in phosphate-buffered saline (PBS) and cultured in full media for a further 48 h. For each virus combination, 3 wells were transduced. At the end of the incubation, cells were washed and permeabilized with 100 μl of reporter cell lysis buffer (Promega) for Luc activity assay or 100 μl of radioimmunoprecipitation (RIPA) buffer (50 mM Tris, 1% NP-40, 1% Sodium Deoxycholate, 0.1% SDS, 150 mM NaCl, 1 mM EDTA, pH 7.5) plus a protease inhibitor cocktail (Sigma) for western blot analysis respectively. The lysed samples can be kept at -80°C until processing.

### In vivo stereotaxic injections

All experiments were carried out in accordance with the Animals Scientific Procedures Act 1986. The animals were housed individually, allowed normal rat chow and drinking water ad libitum, and kept on a 12-hour light/12-hour dark cycle. Briefly, adult male Wistar rats (200~250 g) were anaesthetized with ketamine and medetomidine intramuscularly and their heads mounted securely in a stereotaxic frame. LVV were microinjected stereotaxically into either the DVC including the hypoglossal motor nucleus as before [8] or HIP at the following coordinates: anterior, -4.4 mm; lateral, +3.2 mm; ventral, -2.5 mm from the surface of the dura as described in [29]. A total of six microinjections of viral vectors were made bilaterally for both DVC and HIP injections. In cases where three viral vectors had to be co-injected, their ratio was fixed to 1:1:1 and the total dose was 6 × 10^6 ^infection unit (iu) per rat. In cases where two viral vectors were co-injected, their ratio was fixed to 1:4 and the total dose was also 6 × 10^6 ^iu per rat. The injection rate was 0.5 μl/min and the injection needle was allowed to remain in situ for 5 min before being slowly retracted at the end of each injection. Dox was administered at a concentration of 2 mg/ml supplemented with 5% sucrose in the animals' drinking water as required. Seven days postinjection, rats were terminally anaesthetized (sodium pentobarbital, 100 mg/kg intramuscularly) and perfused through the heart with 0.1 M PBS (pH 7.4). For Luc activity assays, the brain tissue samples were removed and stored at -80°C until processing. After adding PBS buffer (100 μl PBS per 50 mg tissue), each sample was homogenized by sonication for 10 sec on ice, and then centrifuged at 13000 rpm at 4°C. In total, 10 μl of the supernatant at room temperature was used for the Luc activity assay. For nNOS western blot analysis, the brain tissue samples were removed and immediately homogenized with a manual homogenizer in RIPA buffer containing protease inhibitor cocktail. Total protein extracts were then kept at -80°C until further processing.

### Luciferase assay

Luciferase assay was performed with a luciferase assay kit (Promega) in a single-tube luminometer as described [29]. The results are expressed in relative light units (RLU) per well of a 24-well plate for *in vitro *experiments or per rat region for *in vivo *experiments. Data are expressed as the mean ± standard deviation.

### Western blot analysis

The nNOS western blot analysis was carried out as previously described [30]. Briefly, total protein was extracted from homogenized samples, followed by quantification with a BCA protein assay kit (Pierce). 20 μg of total protein per lane were separated on NuPAGE 4-12% Bis-Tris gels (Invitrogen) and transferred to PVDF membranes (Millipore). The membranes were blocked in 5% non-fat dry milk (NFDM) in Tris-buffered saline with 0.1% tween-20 (TBST) for 45 min, and incubated with polyclonal rabbit anti-nNOS antibody (Zymed) at 1:5000 in 3% NFDM-TBST or monoclonal anti-beta-actin antibody (Sigma) at 1:5000 in 1% BSA-TBST overnight. Following incubation with polyclonal swine anti-rabbit immunoglobulins/HRP (Dako) at 1:5000 in 3% NFDM-TBST or polyclonal rabbit anti-mouse immunoglobulins/HRP (Dako) at 1:10000 in 1% BSA-TBST for 90 min, the immunoreactions were detected with an Immun-Star Western chemiluminescent kit (Bio-Rad) and Amersham high performance autoradiography film (GE Healthcare). Scion Image software (Scion Corporation) was used to quantitatively compare the relative blot intensities.

### Northern blot analyses

Total RNA was isolated from rat tissues seven days post-injection using mirVana Isolation Kit (Ambion). Fifteen micrograms total RNA were fractionated on a 15% denaturing polyacrylamide gel and blotted onto Hybond-XL membrane (Amersham). RNA was immobilized by UV crosslinking and baking for 1 hour at 80°C. Hybridization was carried out at 42°C using UltraHyb-Oligo Hybridization buffer (Ambion). Probes were labeled with ^32^P using T4 polynucleotide kinase (New England Biolabs). Membranes were washed twice in 2xSSC, 0.1% SDS and 0.2xSSC, 0.1% SDS at 37°C and exposed to film. Scion Image software (Scion Corporation) was used to quantitatively compare the relative blots intensities.

### Real time RT-PCR

Total RNA was isolated from cell lines and rats using RNAqueous-Micro kit (Ambion) and treated with DNase I. RNA purity was verified by performing PCR on samples not treated with reverse transcriptase. Real-time RT-PCRs were carried out using a DNA Engine Opticon 2 system (MJ Research) and the QuantiTect SYBR Green RT-PCR kit (Qiagen), as described [31]. Expression of target genes was assessed in relation to a housekeeping gene (β-actin) using the comparative Pfaffl method [ratio = (E_target_)^ΔCT target (control-treated)^/(E_ref_)^ΔCT ref(control-treated)^] in each sample [32]. Fold differences against DVC were calculated (n = 5). PCR primers for rat Dicer, Argonaute proteins (Ago), Digeorge syndrome critical region gene 8 (DGCR8), Exportin 5 and Drosha were designed according to sequence information provided by NCBI with the accession numbers: XM_001069041, XM_001058231, NM_001105865, NM_001108789 and NM_001107655 respectively. Among the above, XM_001069041 for Dicer and XM_001058231 for Ago are predicted sequences by automated computational analysis using gene prediction method: GNOMON, supported by mRNA and EST evidence. Primer sequences are detailed in the Additional file 1.

## Results

### Combinations of vectors used in this study

Ten different viral combinations were used here. To simplify the presentation, we used abbreviations. Combinations and abbreviations are presented in Table 1.

**Table 1:** Viral combinations used in this study and their functions.

Abbreviation	Vector combination	Function
**LVVs-miRLuc-neurone**	LV-SYN-Luc + LV-Tretight-GFP-miR30-shRNA/Luc + LV-mCMV/SYN-tTA	Neurone-specific Luc knock-down system. Tet-off transactivator tTA is expressed from LV-mCMV/SYN-tTA which contains the bidirectional amplified SYN promoter to ensure high level of tTA. tTA binds to Tretight promoter in LV-Tretight-GFP-miR30-shRNA/Luc and activates the expression of shRNA/Luc.

**LVVs-miRLuc-glia**	LV-GfaABC_1_D-Luc +LV-Tretight-GFP-miR30-shRNA/Luc +LV-mCMV/GfaABC1D-tTA	Astrocyte-specific Luc knock-down system. Transactivator tTA is expressed from LV-mCMV/GfaABC1D-tTA which contains the bidirectional amplified GfaABC1 D promoter to ensure high level of tTA. tTA binds to Tretight promoter in LV-Tretight-GFP-miR30-shRNA/Luc and activates the expression of shRNA/Luc.

**LVVs-miRLuc-control1**	LV-SYN-Luc + LV-Tretight-GFP-miR30-shRNA/Luc + LV-SYN-WPRE	Control combination used in Luc knock-down experiments for the neurone-specific system. Since there is no tTA expression in this combination, shRNA/Luc will not be expressed. So this combination marked the background expression level of Luc driven by SYN promoter.

**LVVs-miRLuc-control2**	LV-GfaABC_1_D-Luc + LV-Tretight-GFP-miR30-shRNA/Luc + LV-GfaABC1D-WPRE	Control combination used in Luc knock-down experiments for the astrocyte-specific system. Similar as LVVs-miRLuc-control 1, there is no tTA expression in this combination; shRNA/Luc will not be expressed. Therefore this combination marked the background expression level of Luc driven by GfaABC1 D promoter.

**LVVs-miRnNOS-neurone**	LV-Tretight-GFP-miR30-shRNA/nNOS + LV-mCMV/SYN-tTA	Neurone-specific nNOS knock-down system. Mechanism is similar as LVVs-miRLuc-neurone.

**LVVs-miRnNOS-glia**	LV-Tretight-GFP-miR30-shRNA/nNOS + LV-mCMV/GfaABC1D-tTA	Astrocyte-specific Luc knock-down system. Mechanism is similar as LVVs-miRLuc-glia.

**LVVs-miRnNOS-control1**	LV-Tretight-GFP-miR30-shRNA/nNOS + LV-SYN-WPRE	Control combination used in nNOS knockdown experiments for the neurone-specific system. Mechanism is similar as LVVs-miRLuc-control1.

**LVVs-miRnNOS-control2**	LV-Tretight-GFP-miR30-shRNA/nNOS + LV-GfaABC_1_D-WPRE	Control combination used in nNOS knockdown experiments for the astrocyte-specific system. Mechanism is similar as LVVs-miRLuc-control2.

**LVVs-miRnNOS-negative control1**	LV-Tretight-GFP-miR30-shRNA/Luc + LV-mCMV/SYN-tTA	Negative control combination used in nNOS knock-down experiments for the neurone-specific system. Instead of producing shRNA/nNOS it produces shRNA/Luc which shouldn't work on nNOS knockdown.

**LVVs-miRnNOS-negative control2**	LV-Tretight-GFP-miR30-shRNA/Luc + LV-mCMV/GfaABC1D-tTA	Negative control combination used in nNOS knock-down experiments for the astrocyte-specific system. Instead of producing shRNA/nNOS it produces shRNA/Luc which shouldn't work on nNOS knockdown.

### Analyses of the functions of miR30-shRNA/Luc in vitro

**Figure 2:**
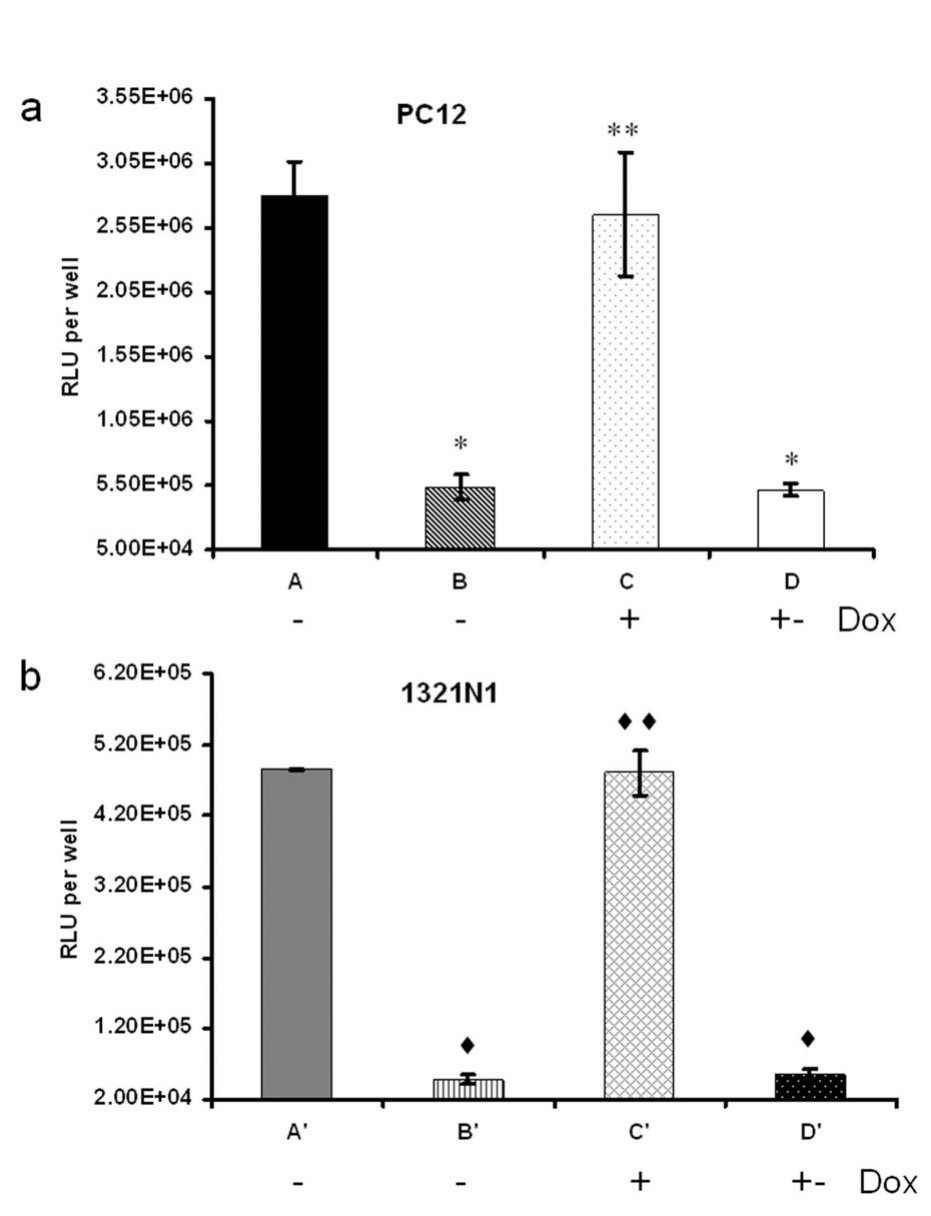
**Analyses of the functions of miR30-shRNA/Luc *in vitro***. (a) LV-mCMV/SYN-tTA and (b) LV-mCMV/GfaABC_1_D-tTA mediated Dox-controllable Luc knock-down in PC12 cells and 1321N1 cells. Dox was used at a concentration of 200 ng/ml. - Dox, cells were cultured in the continuous absence of Dox; + Dox, cells were cultured in the continuous presence of Dox; + - Dox, Dox was administered for 48 h during and after transduction followed by a change to Dox-free medium and culturing for three more days. The results are expressed in RLU per well. A: LVVs-miRLuc-control1; B, C, D: LVVs-miRLuc-neurone A': LVVs-miRLuc-control2; B', C', D': LVVs-miRLuc-glia. *P < 0.01, **P = 0.06, compared with group A. ♦*P *< 0.01, ♦♦*P *= 0.84, compared with group A'. In this and the following figures the error bars represent standard deviation.

First, the effect of miR30-shRNA/Luc was assessed in cell lines. PC12 cells were co-transduced with viral combinations of LVVs-miRLuc-control1 and LVVs-miRLuc-neurone while 1321N1 cells were co-transduced with LVVs-miRLuc-control2 and LVVs-miRLuc-glia. In the absence of Dox, Luc expression from LVVs-miRLuc-neurone [Figure 2(a), group B] was significantly knocked down by 80% as compared to that from LVVs-miRLuc-control1 [Figure 2(a), group A] [5.37 × 10^5 ^± 9.25 × 10^4 ^vs 2.8 ×10^6 ^± 2.67 × 10^5 ^RLU per well; *P *< 0.01] in PC12 cells. Similarly, in 1321N1 cells, Luc expression from LVVs-miRLuc-glia [Figure 2(b), group B'] was significantly reduced by 90% as compared to that from LVVs-miRLuc-control1 [Figure 2(b), group A'] (4.79 × 10^4 ^± 6.54 × 10^3 ^vs 4.85 × 10^5 ^± 1.81 × 10^4 ^RLU per well; *P *< 0.01). The Luc knock-down was completely prevented in the presence of Dox in both PC12 and 1321N1 cells [Figure 2(a), group C vs B and Figure 2(b), group C' vs B']. The inhibitory effect of Dox disappeared after three days of Dox withdrawal [Figure 2(a), group D vs C and Figure 2(b), group D' vs C']. These results demonstrate that bidirectional transcriptionally amplified SYN and GfaABC_1_D promoters provide a sufficient level of tTA to activate the Tretight promoter which then drives the synthesis of GFP-miR30-shRNA/Luc transcript to induce substantial Luc knock-down.

Of note, the astrocytic system LVVs-miRLuc-glia was more effective than the neuronal system, LVVs-miRLuc-neurone. We therefore tested whether this could reflect a higher efficacy of the amplification strategy when applied to the GfaABC_1_D as compared to SYN promoter. To this end, we performed a real time PCR analysis of tTA expression level from LV-mCMV/GfaABC_1_D-tTA in 1321N1 cells and LV-mCMV/SYN-tTA in PC12 cells as compared to LV-GfaABC_1_D-tTA and LV-SYN-tTA respectively. LV-mCMV/GfaABC_1_D-tTA increased tTA expression 14.5 fold as compared to LV-GfaABC_1_D-tTA while LV-mCMV/SYN-tTA increased 7.6 fold as compared to LV-SYN-tTA (data not shown). Thus, higher efficacy of the astrocytic system is likely to reflect a higher level of tTA in glial cells and therefore a higher expression of the gene knock-down cassette.

### Analysis of the effects of miR30-shRNA/Luc in vivo

**Figure 3:**
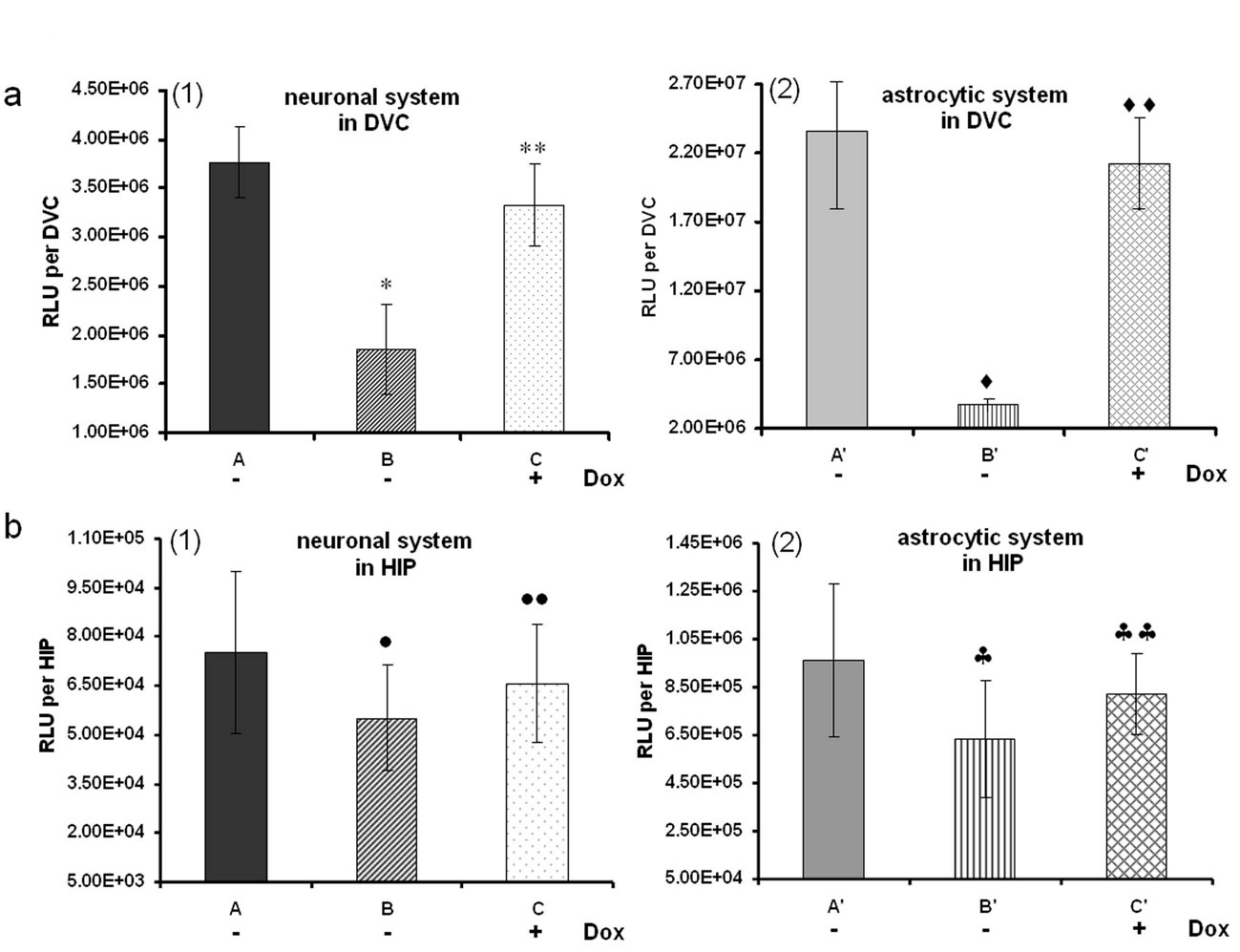
**Analyses of the efficacy of miR30-shRNA/Luc *in vivo *in adult rat brain**. a: (1) LV-mCMV/SYN-tTA and (2) LV-mCMV/GfaABC_1_D-tTA mediated Dox-controllable Luc knock-down in DVC in rats. b: (1) LV-mCMV/SYN-tTA and (2) LV-mCMV/GfaABC_1_D-tTA mediated Dox-controllable Luc knock-down in HIP in rats. Rats in groups A, A', B and B' were not treated with Dox. Rats in groups C and C' drank Dox-containing water post-injection for 7 days. There were 3 rats in each group. A: LVVs-miRLuc-control1; B, C: LVVs-miRLuc-neurone; A': LVVs-miRLuc-control2; B', C': LVVs-miRLuc-glia. *P < 0.01, **P = 0.18, compared with group A. ♦*P *< 0.01, ♦♦*P *= 0.48, compared with group A'. •*P *= 0.12, ••*P *= 0.23, compared with group A. ♠*P *= 0.03, ♠♠*P *= 0.17, compared with group A'.

Next we investigated the effect of miR30-shRNA/Luc *in vivo*. LVVs-miRLuc-neurone, LVVs-miRLuc-glia and the corresponding control vectors were injected into the DVC and HIP of adult rats. As shown in Figure 3a(1), in the absence of Dox, LVVs-miRLuc-neurone (Figure 3a, group B) suppressed DVC neuronal Luc expression by 50% as compared to the level achieved using LVVs-miRLuc-control1 (Figure 3a, group A) (1.85 × 10^6 ^± 4.58 × 10^5 ^vs 3.77 × 10^6 ^± 3.70 × 10^5 ^RLU per DVC, *P *< 0.01). LVVs-miRLuc-glia (Figure 3a, group B') suppressed DVC glial Luc expression even more dramatically (Figure 3a ~85%) as compared to level of expression using LVVs-miRLuc-control2 (Figure 3a, group A') (3.69 × 10^6 ^± 4.54 × 10^5 ^vs 2.37 × 10^7 ^± 5.71 × 10^6 ^RLU per DVC, *P *< 0.01). This was most likely due to a higher potency of LV-mCMV/GfaABC_1_D-tTA as compared to LV-mCMV/SYN-tTA as revealed by real time PCR analysis of tTA expression from our *in vitro *work (see previous section). In both cell types, the knock-down could be completely blocked by administration of Dox into the drinking water [group C in Figure 3a(1); group C' in Figure 3a(2)]. We then tested whether gene knock-down in DVC is indeed cell-specific. To do this, we injected the neurone-targeted gene knock-down system (LV-mCMV/SYN-tTA + LV-Tretight-GFP-miR30-shRNA/Luc) with LVV to express Luc in astrocytes (LV-GfaABC_1_D-Luc) and, conversely, the astrocyte-targeted knock-down system (LV-mCMV/GfaABC_1_D-tTA+ LV-Tretight-GFP-miR30-shRNA/Luc) together with LVV for neuronal Luc expression (LV-SYN-Luc). No knock-down occurred in either case (see Additional file 2) confirming a high degree of cell-specificity of this effect.

Surprisingly, much weaker Luc knock-down was observed using both the neurone-specific and the astrocyte-specific systems, LVVs-miRLuc-neurone and LVVs-miRLuc-glia in HIP compared to DVC [Figure 3b(1), B vs A; Figure 3b(2), B' vs A' ]. Reduction in Luc levels with neuronal system was ~ 27% (5.5 × 10^4 ^± 1.64 × 10^4 ^vs 7.52 × 10^4 ^± 2.48 × 10^4 ^RLU per HIP) and ~ 35% with the astrocytic system (6.32 × 10^5 ^± 2.43 × 10^5 ^vs 9.65 × 10^5 ^± 3.20 × 10^5 ^RLU per HIP). Neither effect was significant (*P *> 0.05), although the trend was clear in both cases.

### nNOS gene knock-down in vitro and in vivo

**Figure 4:**
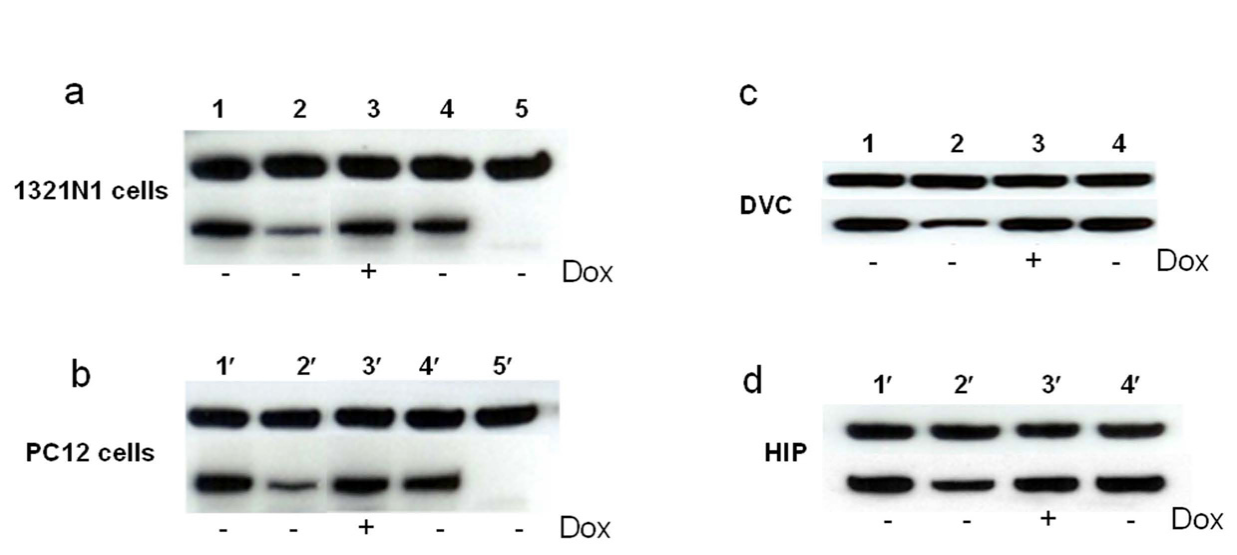
**Western-blot analyses of the functions of miR30-shRNA/nNOS both *in vitro *(a, b) and *in vivo *(c, d)**. a: *In vitro *tests in PC12 cells. - Dox, cells were cultured in the continuous absence of Dox; + Dox, cells were cultured in the continuous presence of Dox; 1: AD-CMV-nNOS + LVVs-miRnNOS-control1; 2, 3: AD-CMV-nNOS+ LVVs-miRnNOS-neurone; 4: AD-CMV-nNOS+ LVVs-miRnNOS-negative control1; 5: mock transfection. b: *In vitro *tests in 1321N1 cells. 1: AD-CMV-nNOS + LVVs-miRnNOS-control2; 2, 3:AD-CMV-nNOS + LVVs-miRnNOS-glia; 4: AD-CMV-nNOS + LVVs-miRnNOS-negative control2 5': mock transfection. c, d: *In vivo *tests in DVC (c) and HIP (d) in rats. Rats in groups 2, 4, were not treated with Dox. Rats in group 3 drunk Dox-containing water post-injection for 7 days. There were 3 rats in each group. 1: mock infection; 2, 3: LVVs-miRnNOS-neurone; 4: LVVs-miRnNOS-negative control2.

While Luc knock-down is technically convenient it is an artificial approach as this gene is not expressed in the mammalian brain. Thus, it was important to demonstrate whether an endogenous gene is as prone to knock-down as an exogenously expressed transcript. We chose nNOS since this protein is abundantly expressed in both DVC and HIP (Figure 4 and [22-25]).

We first confirmed the efficacy of the anti-nNOS construct, LV-Tretight-GFP-miR30-shRNA/nNOS in PC12 and 1321N1 cells. An adenoviral vector (AD) AD-CMV-nNOS was used to induce high level of nNOS expression in both PC12 and 1321N1 cells since there are almost no endogenous nNOS expression in these two cell lines (unpublished observation). As shown in Figure 4 in the absence of Dox, both LVVs-miRnNOS-neurone and LVVs-miRnNOS-glia markedly knocked down nNOS in PC12 cells (~ 69% reduction in optical density, treatment 2 vs treatment 1, Figure 4a) and 1321N1 (~ 82% reduction, treatment 2 vs treatment 1, Figure 4b). This nNOS knock-down effect could be prevented by Dox (treatment 3 in Figure 4a and Figure 4b). It is important to note that anti-Luc construct, LV-Tretight-GFP-miR30-shRNA/Luc (treatment 4 in Figure 4a and Figure 4b), was without effect in either cell line, indicating that the nNOS knock-down was sequence-specific.

A substantial nNOS knock-down (~ 55% reduction) (treatment 2 vs treatment 1, Figure 4c) was observed when LVVs-miRnNOS-neurone was injected into DVC *in vivo *and consistent with the *in vitro *data this knock-down could be fully prevented by Dox (treatment 3). However, similar to the experiment with Luc, the nNOS knock-down in HIP (~ 35% reduction; treatment 2 vs treatment 1, Figure 4d) was noticeably weaker. Again, the anti-Luc construct LV-Tretight-GFP-miR30-shRNA/Luc (treatments 4 in Figure 4c and Figure 4d) did not trigger nNOS knock-down.

### Analysis of miRNA processing enzyme expression in DVC and HIP

**Figure 5:**
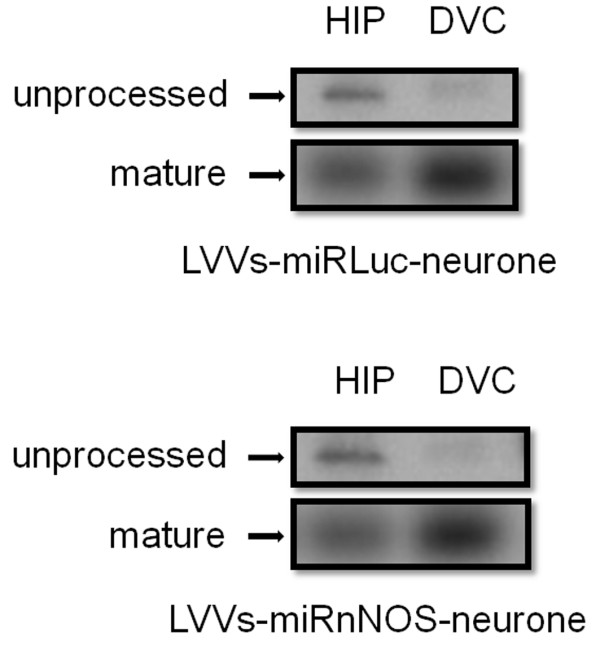
**Northern blot analyses to assess the processing of RNAi in HIP and DVC**. Total RNA samples were isolated from rats injected with LVVs-miRLuc-neurone (upper half) or LVVs-miRnNOS-neurone (lower half) into HIP and DVC. Blots were probed for either Luc or nNOS shRNA transcripts.

**Figure 6:**
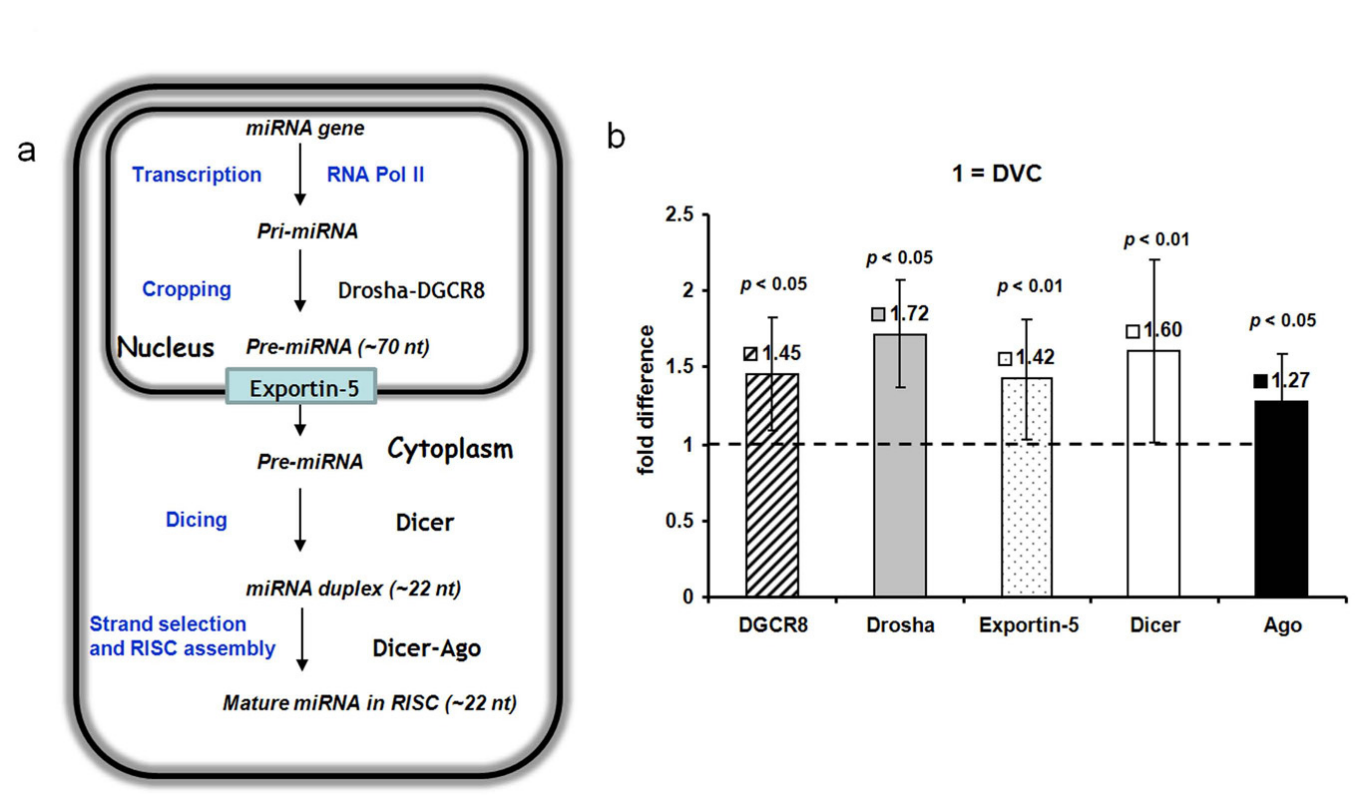
**Model for miRNA biogenesis and real time PCR analysis of the five key enzymes implicated in miRNA biogenesis. a**: Model for miRNA biogenesis. miRNA genes are transcribed by Pol II to generate the primary transcripts (pri-miRNAs). The initiation step (cropping) is mediated by the Drosha-DGCR8 complex. The product of the nuclear processing is ~ 70-nt pre-miRNA. This structure can serve as a signature motif that is recognized by the nuclear export factor Exportin-5. Pre-miRNA constitutes a transport complex together with Exportin-5 and its cofactors. Upon export, the cytoplasmic RNase III Dicer participates in the second processing step (dicing) to produce miRNA duplexes (~ 22-nt). The duplex is recognized by the PAZ domain of the Ago protein and incorporated into RISC. Usually one strand is selected as the mature miRNA, whereas the other strand is degraded. **b**: Real time PCR analyses of the expression levels of DGCR8, Drosha, Exportin-5, Dicer and Ago in DVC and HIP in rats (n = 5). The value for DVC was set as one.

We were surprised to find that the same knock-down cassettes driven by the same targeting systems behaved differently when applied to DVC as compared to HIP. To examine whether the different RNAi efficiency in DVC and HIP is caused by different processing of RNAi, we performed northern blotting analysis to assess the ratio between mature-RNAi and precursor-miR30-RNAi in these two regions. We found higher ratio for both Luc RNAi and nNOS RNAi from DVC relative to that from HIP (Figure 5). The ratios of mature RNAi to unprocessed from LVVs-miRLuc-neurone injected rats are ~1:1 in HIP and ~ 18:1 in DVC respectively. With LVVs-miRnNOS-neurone, the ratios are ~ 1.3:1 in HIP and ~ 23.5:1 in DVC respectively. We then hypothesized that variations in the composition of the RNAi machinery are a likely cause of this site-specific difference in RNAi processing. To test this idea, we conducted real time RT-PCR analysis of 5 key enzymes implicated in miRNA biogenesis, namely Drosha, DGRC8, Exportin-5, Dicer and Argo (Figure 6a). All five genes tested were found in both DVC and HIP and their expression in HIP was marginally higher than in DVC. Thus, lower gene knock-down efficacy in HIP is unlikely to be related to regional differences in the availability of the key components on miRNA pathway between DVC and HIP (Figure 6b).

## Discussion

This study was designed to address two questions. Firstly, we wanted to test the efficacy of Pol II-based cell-type specific and Dox-controllable gene knock-down in the brain. Secondly, we wanted to find out whether the same knock-down system should be expected to operate with equal effectiveness in different parts of the CNS. The reason for posing this question is that the lower parts of the CNS, such as medulla oblongata, differ from the higher brain in many respects, including their cellular composition, embryonic origins and gene expression patterns. Neither of these questions has been addressed previously. Moreover, we wanted to assess the effect at the protein level rather than mRNA content since it remains unclear how these two readouts of gene expression are affected by different RNAi constructs, many of which are also thought to operate via translational repression rather than through mRNA degradation.

Lentiviral systems developed in the course of this study enable tight Dox-controllable and cell-specific miR30-based RNAi gene knock-down. Using the Tet system in these designs bring additional benefits because it not only allows switching off the knock-down effect but also simplifies re-targeting of the knock-down vector to different cell types, provided that a sufficiently cell type specific promoter is available. For the Tet system to operate it is essential to achieve high levels of Tet transactivator expression. Mammalian cell-specific promoters are seldom sufficiently powerful, so we have used the previously validated bidirectional TSTA [26] to enhance two such weak promoters here: the SYN and GfaABC_1_D. Interestingly, the degree of enhancement achieved using the same bidirectional TSTA strategy was higher for GfaABC_1_D as indicated by a much higher increase in tTA transcription from enhanced GfaABC_1_D compared to enhanced SYN (see results *Analyses of the functions of miR30-shRNA/Luc in vitro*). This correlated with the overall higher efficacy of gene knock-down achieved with the astrocyte-targeted system both *in vitro *and *in vivo *than the neuronal specific system.

Our experiments show that at least in the DVC we can expect a very significant knock-down of the target protein (~ 55%) with our approach. The efficacy of knock-down seems to correlate with the power of the vector used to express the tTA, making astrocytic system slightly more potent than the neuronal one. It is clear that there is no cross-talk between neurone- and astrocyte-targeted knock-down since when Luc is placed in the phenotype not targeted by the knock-down vectors there is no decrease in expression (see Additional file 2). This also strongly argues against the involvement of any non-specific factors such as local interferon induction. Removal of the knock-down response by Dox (Figures 2, 3 and 4) is another argument for the specific nature of this effect.

Both Pol II and Pol III promoters have been used to express shRNA for RNAi [33]. Unlike most small cellular RNAs, miRNAs are primarily transcribed by Pol II, which may reflect a need for more moderate, regulatable and cell-specific expression. In the context of this study, the Pol II driven production of miRNA-based shRNA allows cell-specific knock-down and exogenous control using the Tet-system. In addition, Pol II promoters in miRNA-based RNAi systems have no strict requirements for the transcriptional start site and termination signal so theoretically various Pol II promoters can be used [2,9,34]. Insertion of a GFP reporter gene upstream of miR-shRNAs is thought to have at least two advantages. Firstly, this allows monitoring of the shRNA production in individual cells. Indeed we have noticed GFP expression in our experiments (data not shown). Secondly, the expression of miR-shRNAs and protein mRNAs in a monocistronic transcript could lead to both effective processing of the miR-shRNAs and translation of protein from the mRNA [15,35,36].

It is important to realize that when Luc was used as a target, vectors to express Luc and the knock-down LVVs were applied together as a mixture. While they should have been internalized by the same population of cells in the target area it is possible that some cells would only take in one or two vectors, but not all three. Therefore, a possibility is that some of the residual Luc expression comes from those cells where the binary knock-down system was absent or did not assemble in full (for example, no LVV to express Tre was internalized). When nNOS is chosen as a target, the protein can only come from the cells which endogenously express it in the target area. It is likely that the level of most endogenous genes will be less than those expressed exogenously by a LVV. This could be one of the reasons why nNOS knock-down was evident not only in DVC (as was the case for Luc) but also in HIP (Figure 4c and 4d) although Luc knock-down was only evident as a trend in HIP (Figure 3b).

To our knowledge, this is the first study to evaluate the different efficacies of miR30-based RNAi system among different regions in the brain *in vivo*. At present we do not know what accounts for this difference. We hypothesized that lower efficacy of the tested constructs in HIP compared to DVC was a result of a lower level of expression of the components involved in the RNAi pathway. However, real-time RT-PCR analysis of Dicer, Argonaute, DGRC8, Exportin 5 and Drosha revealed slightly higher levels of all of these transcripts in HIP than in DVC. Hence, other untested or unknown components must account for the difference. RNAi is a dynamic process and its machinery depends on multiple components with only poorly characterized expressions patterns [37]. Moreover, novel components involved in posttranscriptional gene silencing are very likely to emerge. Accumulating evidence indicates that many miRNAs show distinct expression patterns in an organ or tissue-specific way [37-39]. Our constructs, following the design of Stegmeir *et al*. used flanking and loop sequences from an endogenous miR30 [37]. A recent study uncovered 44 miRNAs which exhibited marked differences in the level of expression between spinal cord, cerebellum and hippocampus in the adult mouse [40]. For example, miR-195, miR-497, and miR-30b were found to be enriched in the cerebellum whereas miR-218, miR-221, miR-222, miR-26a, miR-128a/b, miR-138 and let-7c were highly expressed in the HIP. Unfortunately, the DVC was not studied specifically. It is not impossible that in the brain there are not only regional differences in the levels of expression of the miRNAs but also of some other factors which favour the the production and maturation of specific miRNA. If that was the case, processing of knock-down constructs built using elements of some miRNAs could be more efficient in some parts of the brain than in others as we have found herein. This should be kept in mind for loss-of function studies in the rat brain.

In summary, we have demonstrated that efficient, cell-specific and Dox-controllable gene knock-down can be achieved in the rat brain although the potency of the knock-down using the same construct may differ in different parts of the CNS. This efficient gene silencing system will be a valuable resource for basic gene function study and potentially, for the development of gene-based therapeutics of the CNS. While this manuscript was under review, a study where we have used the system for neurone-specific knock-down of nNOS was published [41], demonstrating the practical value of this approach. In that study we demonstrated that nNOS plays a key role in several pathological processes triggered in motor neurones by axotomy. Critically, the knock-down in that experiment was performed in the same brainstem area where, as we show here, this type of RNAi has proven to be most effective. Commercial availability of miRNA-like hairpins might create a feeling that any such construct may be used with the same effect in any part of the brain. This study therefore alerts the readers to avoid such an assumption when planning loss-of function studies in the rodent brain. It also illustrates the fact that more research is needed to perfect and optimise the current strategies for RNAi-mediated gene knock-down *in vivo*.

## Conclusions

**S**elective gene knock-down in subsets of brain cells is achievable; however, there are some presently unknown regional factors which affect either the processing of miRNA-based cassettes or their potency for gene silencing.

## Authors' contributions

BL was responsible for experimental design and completion of the bulk of the laboratory work presented in this article and writing of the manuscript. HX did the western blot analysis. SK designed the study and participated in all aspects of the work. JFRP helped to coordinate the study. All authors have read and approved the final manuscript.

## Supplementary Material

Additional file 1 **Table S1**. Primers used for real time PCR. Primer sequences used in the real time PCR analysis of five key enzymes implicated in miRNA biogenesis including DGCR8, Drosha Exportin 5, Dicer and Argonaute proteins in rats. Click here for file

Additional file 2 **Figure S1 Neurone- and astrocyte-targeted knock-down is cell-type specific**. LV-mCMV/SYN-tTA and LV-mCMV/GfaABC_1_D-tTA mediated luciferase knock-down is cell-type specific. a: LV-mCMV/GfaABC_1_D-tTA controlled miR30-shRNA/Luc didn't knockdown Luc expression in neurones. b: LV-mCMV/SYN-tTA controlled miR30-shRNA/Luc didn't knockdown Luc expression in glia. A: LVVs-miRLuc-control1; B: LV-SYN-Luc + LV-Tretight-GFP-miR30-shRNA/Luc + LV-mCMV/GfaABC_1_D-tTA. A': LVVs-miRLuc-control2; B': LV-GfaABC_1_D-Luc + LV-Tretight-GFP-miR30-shRNA/Luc + LV-mCMV/SYN-tTA. Click here for file
