# Supplementary material for: Cell- and region-specific miR30-based gene knock-down with temporal control in the rat brain
Source: BMC Mol Biol. 2010 Dec 6;11:93. doi: 10.1186/1471-2199-11-93 (PMC3047298; doi:10.1186/1471-2199-11-93)
Supplement: Additional file 1 — Table S1. Primers used for real time PCR. Primer sequences used in the real time PCR analysis of five key enzymes implicated in miRNA biogenesis including DGCR8, Drosha Exportin 5, Dicer and Argonaute proteins in rats. [file 1471-2199-11-93-S1.DOC]

**Table S1 Primers used for real time PCR**

| **Gene name** | **NCBI accession number** | **Primer sequences** |
| --- | --- | --- |
| β-actin | NM_031144 | forward: agccatgtacgtagccatc  reverse: accctcatagatgggcaca |
| DGCR8 | NM_001105865 | forward: cgcaatcgttcaaagagaca  reverse: tcttgggagcacagagacct |
| Drosha | NM_001107655 | forward: actcggaggtgttcgatgtc  reverse:aaagtggtccgtcgttcatc |
| Exportin 5 | NM_001108789 | forward: aagtggcatgtcatcaacca  reverse:acatcctcaccagctgttcc |
| Dicer | [XM_001069041](http://www.ncbi.nlm.nih.gov/entrez/viewer.fcgi?db=nuccore&val=109479875) | forward: cccagaaacctgtcaaagtg  reverse: gtcttctttatccaccctgc |
| Argonaute proteins | [XM_001058231](http://www.ncbi.nlm.nih.gov/entrez/viewer.fcgi?db=nuccore&val=109477238) | forward: atctccaaggatgcagggatg  reverse: aacatgggctccacgctgt |
